# Oleomargarine Again

**Published:** 1881-07

**Authors:** 


Oleomargarine Again.—From the first we have regarded this product as a contribution of chemical science to domestic economy, in the interests of the human family. Were it not for motives of trade and the fears of competition on the part of dairymen, there would have been but little question on the subject. The case is one of selfishness and prejudice arrayed against the interests of the people, the poorer classes more particularly. Only, let the article be sold for what it is and not for genuine butter. Impartial analysis has never yet condemned it. Very recently the New York Board of Health, being urged to take action against its use, referred it to Prof. Chandler, State Chemist, for examination and analysis. His report will be likely to stand as a permanent verdict. He says it is superior to lower grades of dairy butter; that there is nothing objectionable in the material, and as there is nothing unwholesome in olemargarine, he sees no need of legislation to protect the public health.—*Pacific Medical and Surgical Journal.*

